# Association between acromial morphology, scapular control, ultrasonographic impingement and non‐surgical treatment outcome in patients with isolated subacromial pain syndrome

**DOI:** 10.1002/ksa.70424

**Published:** 2026-05-20

**Authors:** Adam Witten, Kristian Thorborg, Per Hölmich, Mikkel Bek Clausen, Kristoffer Weisskirchner Barfod

**Affiliations:** ^1^ Sports Orthopedic Research Center – Copenhagen (SORC‐C), Department of Orthopedic Surgery Copenhagen University Hospital Amager‐Hvidovre Hvidovre Denmark; ^2^ Department of Midwifery, Physiotherapy, Occupational Therapy and Psychomotor Therapy, Faculty of Health University College Copenhagen Copenhagen Denmark; ^3^ Department of Orthopedic Surgery Section for Sports Traumatology, Copenhagen University Hospital ‐ Bispebjerg and Frederiksberg Copenhagen Denmark

**Keywords:** acromion, impingement, rotator cuff, subacromial, ultrasonography

## Abstract

**Purpose:**

The aim of this study was to investigate whether acromial morphology, scapular control, and ultrasonographic impingement were associated with treatment outcome from three months of non‐surgical treatment in patients with subacromial pain syndrome (SAPS).

**Methods:**

Patients were recruited consecutively from an orthopaedic outpatient clinic. All patients were systematically investigated for SAPS and for the presence of concomitant diagnoses by orthopaedic specialists using predefined standardised criteria. All patients diagnosed with SAPS were referred for 3 months physiotherapy. The treatment outcome was defined as the change in the Shoulder Pain and Disability Index (SPADI) from pre‐ to post‐treatment. Acromial morphology was evaluated on standardised outlet view radiographs, scapular control was evaluated with the scapular assistance test and ultrasonographic impingement was evaluated using a reliable method. The association between each of the pathophysiological factors and treatment outcome was investigated with linear regression analyses.

**Results:**

A total of 69 patients were included in the analysis for scapular control, 62 patients for ultrasonographic impingement, and 45 patients for the radiographic analysis. Eight patients were lost to follow‐up. Acromial morphology was associated with poorer treatment outcome in both the adjusted and unadjusted linear regression models. The adjusted model predicted a change in SPADI of 1.8 points per degree of increased acromial curvature (more hook‐shaped acromion). There was no association between scapular control or ultrasonographic impingement and change in SPADI.

**Conclusion:**

In patients with isolated SAPS, increasing acromial curvature (more hook‐shaped acromion) was associated with poorer non‐surgical treatment outcome, while ultrasonographic impingement and scapular control were not. This indicates that acromial morphology may be a prognostic factor for non‐surgical treatment outcome, but future research is needed to confirm the findings.

**Level of Evidence:**

Level II, prognostic study.

**Abbreviations**
ASA score: American Society of Anesthesiologists
BMI: body mass index
CAC: Copenhagen Acromial Curve classification
MCID: minimal clinically important difference
NPRS: numeric pain rating scale
NSAIDs: non‐steroidal anti‐inflammatory drugs
SAPS: subacromial pain syndrome
SAT: scapula assistance test
SPADI: Shoulder Pain and Disability Index

## INTRODUCTION

Subacromial pain syndrome (SAPS) is a clinical diagnosis characterised by pain and impaired shoulder function leading to reduced quality of life [11, 16, 27, 43, 50]. Patients with SAPS have been reported to miss more than 5 work weeks within 6–12 months and account for the highest cumulative healthcare costs among shoulder disorders, with an average cost of approximately €30,000 per patient within 6 years [10, 39, 44].

It is recommended that patients with SAPS are offered a structured physiotherapy programme containing shoulder strengthening and scapular control exercises as first‐line treatment, which may be supported by subacromial corticosteroid injections and non‐steroidal anti‐inflammatory drugs (NSAIDs) if deemed necessary [18, 42]. However, only about half of patients achieve satisfactory outcomes from non‐surgical treatment [2, 12, 13, 22]. Currently, there is no method to identify patients who benefit satisfactorily.

The aetiology of SAPS is not fully understood [14]. Several pathophysiological factors have been proposed as potential contributors [5, 17, 26, 30, 47]. However, the association between pathophysiological factors and treatment outcome has only been investigated sparsely [31, 45], and there is currently no evidence to support treatment stratification based on such factors. Acromial morphology, poor scapular control and impingement of the subacromial structures represent some of the key pathophysiological factors that have been associated with SAPS [23, 32, 33, 38, 46]. These pathophysiological factors are variably expressed in patients with SAPS, and may account for some of the variation in treatment outcomes observed between patients [1, 4, 14, 17, 47]. It is reasonable to assume that acromial morphology cannot change in response to non‐surgical interventions. Conversely, it seems plausible that patients with poor scapular control are more likely to respond favourably to an exercise‐based physiotherapy programme, through the prospect of regaining improved scapulothoracic control. Impingement is a poorly understood phenomenon that is hypothesised to arise from various factors, including both structural (hook‐shaped acromion) and dynamic (muscular imbalance) factors. As such, these factors that contribute to impingement may have independent or combined effects on outcome after non‐surgical treatment.

Identifying pathophysiological factors associated with treatment outcome could be an important first step towards developing a more individualised treatment approach for patients with SAPS. Such insight could lead to improved decision‐making in the treatment of SAPS, improved use of healthcare resources, and thus, ultimately, help mitigate the large socio‐economic impact of SAPS.

The aim of this study was to investigate whether acromial morphology, scapular control, and ultrasonographic impingement were associated with treatment outcome from three months of non‐surgical treatment in patients with subacromial pain syndrome (SAPS). We hypothesised that acromial curvature, scapular assistance test and ultrasonographic impingement would be associated with outcome from 3 months of non‐surgical treatment.

## MATERIALS AND METHODS

### Ethics

The study was registered and approved by the Regional Scientific Ethical Committee, Copenhagen Region (Reference no.:H‐19025712). The protocol was uploaded to ClinicalTrials.gov (NCT05549674) before the study commenced.

### Study design

In this prospective study, patients with isolated SAPS were consecutively recruited from the outpatient clinic, Arthroscopic Section, Orthopaedic Department, Copenhagen University Hospital Hvidovre, Denmark, between 1 September 2020 and 31 December 2022.

All participants were referred to a 3‐month structured physiotherapy programme delivered in a municipal setting, with a focus on shoulder strengthening and scapular control exercises. Prior to initiation of physiotherapy, patients underwent evaluation with shoulder outlet‐view radiographs, shoulder ultrasound examination, and a modified scapula assistance test to assess acromial morphology, ultrasonographic impingement, and scapular control. These pathophysiological factors were subsequently analysed for their association with changes in the Shoulder Pain and Disability Index (SPADI) following 3 months of structured physiotherapy.

The present study forms part of a larger investigation of patients with SAPS. The study population overlaps with that of previously published studies [48, 49]. While portions of the demographic and ultrasonographic data have been reported in earlier cross‐sectional analyses, findings related to acromial morphology, the scapula assistance test and the prospective analyses presented here have not been published previously.

### Participant selection

Eligibility screening was conducted in the outpatient clinic by orthopaedic shoulder specialists. It comprised a clinical examination with 17 standardised and protocolized physical tests (Supporting Information: Appendix), an ultrasound examination of the rotator cuff and long head biceps tendon, glenohumeral radiographs (anterior‐posterior and outlet views), and acromioclavicular joint radiographs, to diagnose SAPS and exclude patients with other pain‐contributing diagnoses (Table 1).

**Table 1:** Definition of exclusion criteria.

Exclusion criteria	Definition
Frozen shoulder	Flexion and external rotation ROM decreased >25°, with >50% loss of external rotation ROM
Major shoulder instability	Pos. apprehension‐relocation test or surprise test or Jerk test
Minor shoulder instability	Pos. Castagna's test
SLAP	Pos. O'Brien's test
Biceps tendon pathology	Pos. Speed's test and long head biceps tendon palpation pain (bicipital groove) or long head biceps tendon rupture
Acromioclavicular osteoarthritis	Pos. cross‐over test, acromioclavicular joint palpation pain and radiological signs of osteoarthritis
Full‐thickness rotator cuff tear	Communication between glenohumeral joint and subacromial space (verified with ultrasonography or MRI)
Calcified tendinopathy	>5 × 5 mm calcification on ultrasound or X‐ray
Glenohumeral osteoarthritis	Kellgren‐Lawrence score of 3 or 4 on frontal and sagittal plane radiographic views
Cervical pathology	Cervical pain and positive Spurling test (foramen compression test)
Systemic musculoskeletal or inflammatory joint disease	E.g., Rheumatoid arthritis

*Note*: Partial rotator cuff tears (no communication between glenohumeral joint and subacromial space) were not considered an exclusion criterion.

Abbreviations: MRI, magnetic resonance imaging; ROM, range of motion; SLAP, superior labral anterior to posterior.

### Inclusion criteria

SAPS diagnosis: at least three out of five positive tests from the following: Hawkins' test, Neer's test, Jobe's test, Painful arc and External rotation resistance test, and insidious onset of shoulder pain.

### Exclusion criteria

No sign of other pain‐contributing diagnosis (Table 1), insufficient proficiency in Danish, no e‐mail address, terminal illness or severe medical illness (American Society of Anesthesiologists [ASA] score higher than or equal to 4), previous surgery, fracture or radiotherapy in the affected shoulder region, subacromial corticosteroid injection during the past three months, baseline examination not possible before initiation of physiotherapy.

### Physiotherapy

All patients were referred to three months structured physiotherapy in a municipal setting. Treatment included shoulder strengthening and scapular control exercises performed with elastic bands or light hand weights. It was recommended that patients performed such exercises at least twice a week for a combined duration of at least 45 min. The guiding principle was home‐based exercises combined with structured supervision and feedback during scheduled sessions at the rehabilitation centres. The programme was tailored to each patient's initial shoulder function and symptom development throughout the rehabilitation period, allowing for adjustments in exercise type, load and frequency. Physiotherapy was planned to be initiated within 2 weeks.

### Analgesia

Patients were allowed to self‐administer NSAIDs and paracetamol as needed. None received oral corticosteroids or additional analgesia.

### Outcomes

The primary outcome was change in SPADI from baseline to 3 months follow‐up. SPADI is a patient‐reported outcome measure designed to evaluate shoulder pain and disability. It ranges from 0 (best) to 100 (worst), comprising five pain items and eight disability items. The Danish version of SPADI has demonstrated good reliability [8]. SPADI was distributed via e‐mail.

### Individual pathophysiological factors

The investigated pathophysiological factors and their method of evaluation are presented in Table 2.

**Table 2:** Pathophysiological factors evaluated at the baseline examination.

Pathophysiological factor	Method of evaluation	Outcome
Acromial morphology	Radiography (outlet view)	Degrees
Impingement	Ultrasonography	Present/not present
Scapular control	Scapula assistance test	Positive/negative

The radiographic measurements, the ultrasound assessment and the modified scapula assistance test were performed by the first author (orthopaedic shoulder specialist).

### Acromial morphology

**Figure 1:**
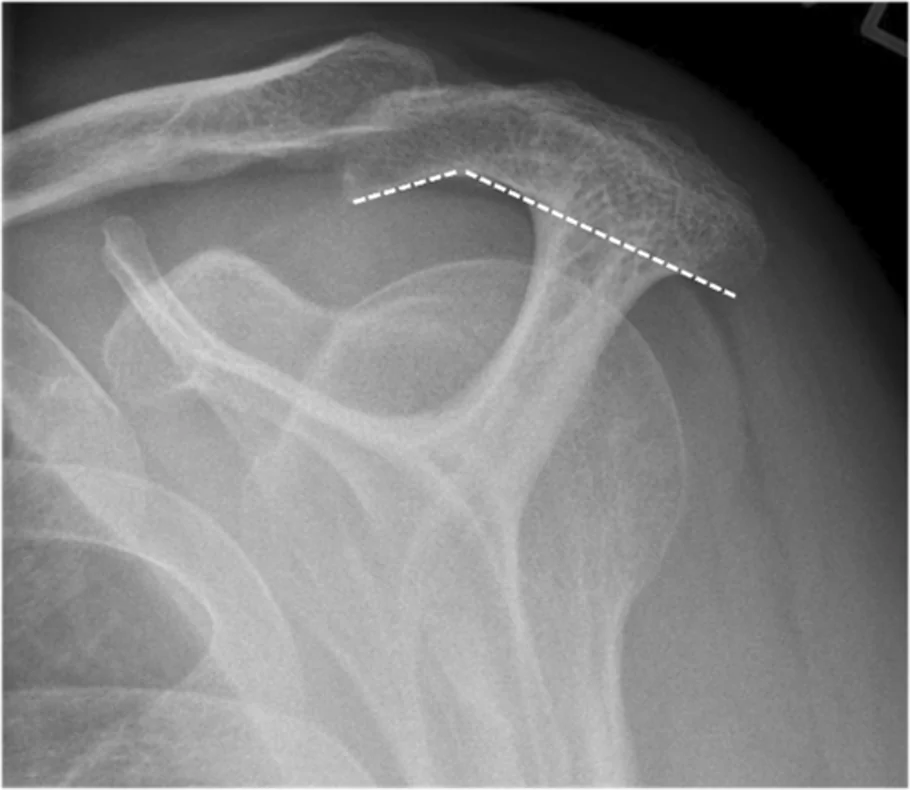
Evaluation of acromial morphology using the Copenhagen Acromial Curve classification.

Acromial morphology was evaluated on radiographs (outlet views) with the Copenhagen Acromial Curve classification (CAC) [28]. The measurement of CAC is performed by drawing two lines. The first line is drawn from the most posterior point of the acromial undersurface to its highest point. The second line is drawn from the most anterior aspect of the acromial undersurface to its highest point. The CAC is the angle between these two lines (Figure 1). The CAC classification has been found to be the most reliable method for classifying acromial morphology [28]. Outlet views meeting the quality standard were considered for the radiographic analyses. The quality standard adhered to the Copenhagen Outlet View Criteria, a method designed to ensure proper alignment, minimising malrotation and misalignment in outlet view radiographs [28]. Radiographs not meeting the Copenhagen Outlet View Criteria were excluded from the analyses.

### Ultrasonographic impingement

**Figure 2:**
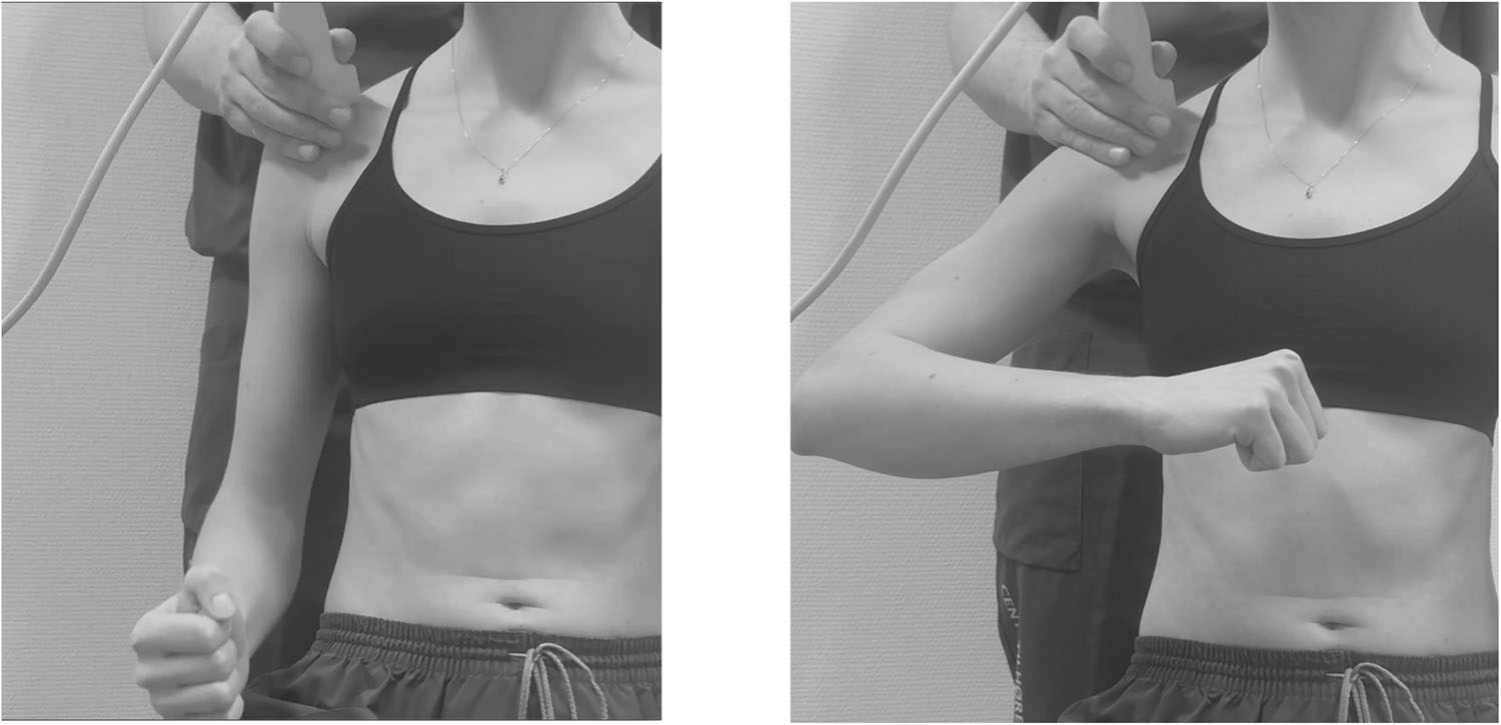
Evaluation of ultrasonographic impingement. Start position (left) and end position (right).

Ultrasonographic impingement was evaluated with a reliable and validated method that has demonstrated a high intrarater reliability (kappa = 0.96) and interrater reliability (kappa = 0.82) [24]. The patient was sitting on a chair with the shoulder in neutral position and the elbow flexed to 90°. The examiner placed the ultrasound transducer at the anterolateral acromion, visualising acromion, the supraspinatus tendon, and the subacromial bursa with a longitudinal view. The patient was instructed to slowly elevate the arm while internally rotating the shoulder, with the transducer held in place (Figure 2). Ultrasonographic impingement was defined as visual bulging of the subacromial bursa against the acromion and rated as present/not present [24]. Ultrasonography was performed on a Hitachi Arietta V70 Diagnostic Ultrasound System (Hitachi Medical Systems, Steinhausen, CH.).

### Scapular control

**Figure 3:**
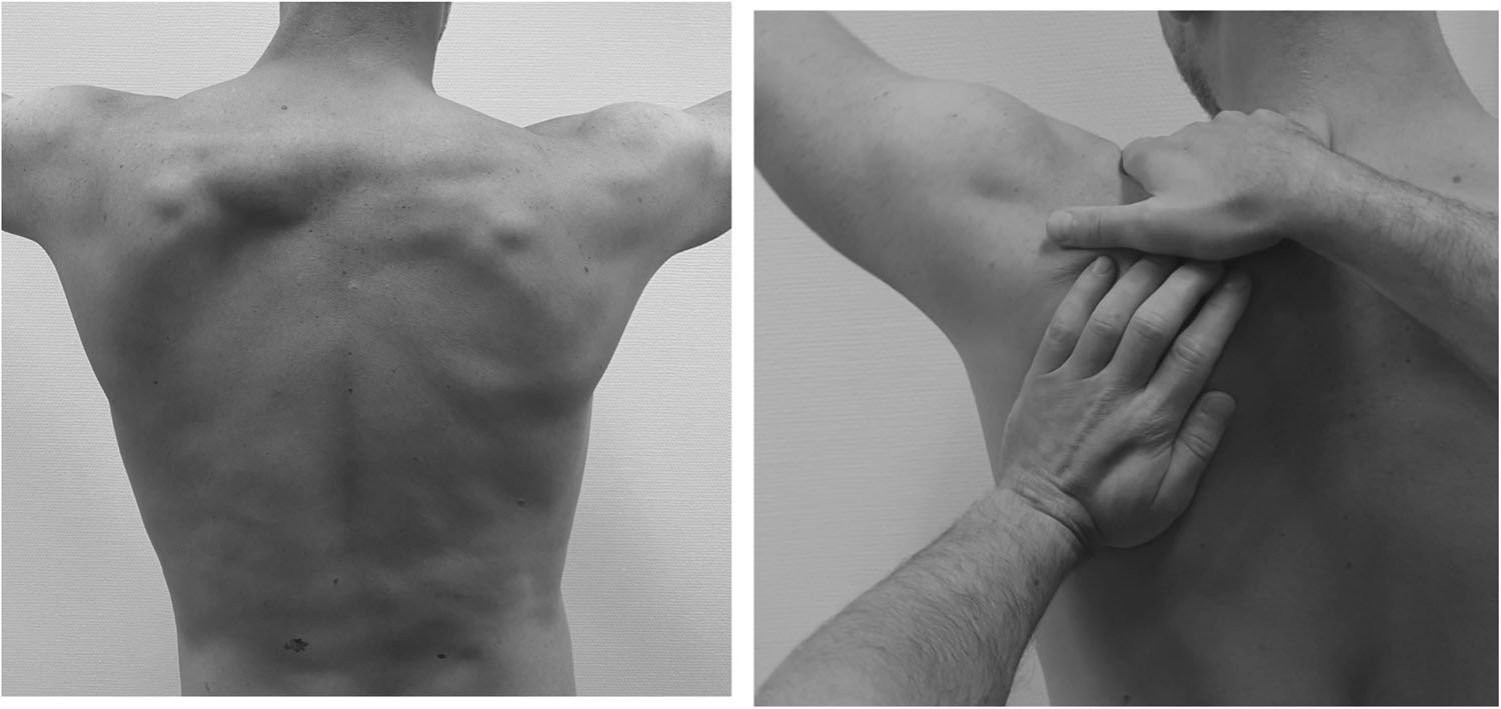
Example of shoulder scapular dyskinesis (left). Examiner manually facilitating a normal scapular movement (right).

Scapular control was evaluated with a modified version of the scapula assistance test (SAT) [9, 23, 25, 29, 36]. The patient was instructed to elevate the arm in the scapular plane as far as possible, while rating the level of shoulder pain using the numeric pain rating scale (NPRS). The patient was then instructed to elevate the arm again, with the examiner manually facilitating a normal scapular movement: a smooth coordinated upward and external scapular rotation, and posterior tilt, without protruding of the scapular borders (winging) (Figure 3). The test was considered positive if the patient reported less pain (at least 2 on the NPRS, or from 1 to 0) or could elevate the arm at least 10° more during the SAT. Originally, the SAT does not include an assessment of range of motion (ROM). The rationale for including a ROM assessment was based on the expectation that many patients would not be able to fully elevate their arm because of too severe shoulder pain. This modified method for evaluating SAT was investigated before the inclusion of patients. Between two raters, the overall agreement for this modified method was 82% (kappa = 0.61) (Supporting Information: Appendix).

### Patient demographics

The following background characteristics were obtained: age, body mass index (BMI), symptom duration and handedness.

### Sample size consideration

Participants were recruited as a convenience sample as part of a larger systematic investigation of patients with SAPS. There were no prior studies to inform a sample size calculation in this exploratory study. A sample size calculation shows that, in order to investigate the contributions of acromial morphology, scapular control, and ultrasonographic impingement to the outcome after three months of non‐surgical treatment, using separate multivariable linear regression models, with a beta level of 0.20 (80% power) and a significance level of 0.05, a total of 55 participants are required to detect a moderate‐to‐large effect size (Cohen's *d* = 0.77) in each model.

### Statistics

Multivariable linear regression analyses were run to estimate the association between acromial morphology, SAT and impingement with the change in SPADI from baseline to after three months of non‐surgical treatment. Age, gender, BMI, symptom duration, and SAPS in the dominant shoulder were evaluated as potential confounders by comparing unadjusted exposure estimates to adjusted exposure estimates for each potential confounder in a separate model. If the difference between the unadjusted exposure estimate and the adjusted exposure estimate was considered to be of a clinically relevant size, the confounder was included in the adjusted model (Supporting Information: Appendix). Symptom duration was a confounder for acromial morphology, with longer symptom duration associated with poorer outcomeand thus, added to the adjusted model for the analysis regarding acromial morphology. There were no confounders for the analyses regarding SAT or impingement. All exposure estimate analyses are found in the appendix. The adjusted models showed linearity as assessed by partial regression plots and a plot of studentized residuals against the predicted values. There was homoscedasticity, as assessed by visual inspection of a plot of studentized residuals versus unstandardised predicted values. The assumption of normality was met, as assessed by a Q‐Q Plot. There was one outlier in the data set (more than three standard deviations from the mean). The outlier was kept in the data set, as a sensitivity analysis determined that the results were not skewed by this. Bonferroni corrections were not applied, and the findings should therefore be regarded as exploratory. Missing data were not imputed. All analyses were conducted using available data only. The statistical plan and the analyses were performed with support from a medical statistician. All analyses were run in IBM SPSS Statistics for Windows, Version 28.0.

## RESULTS

**Figure 4:**
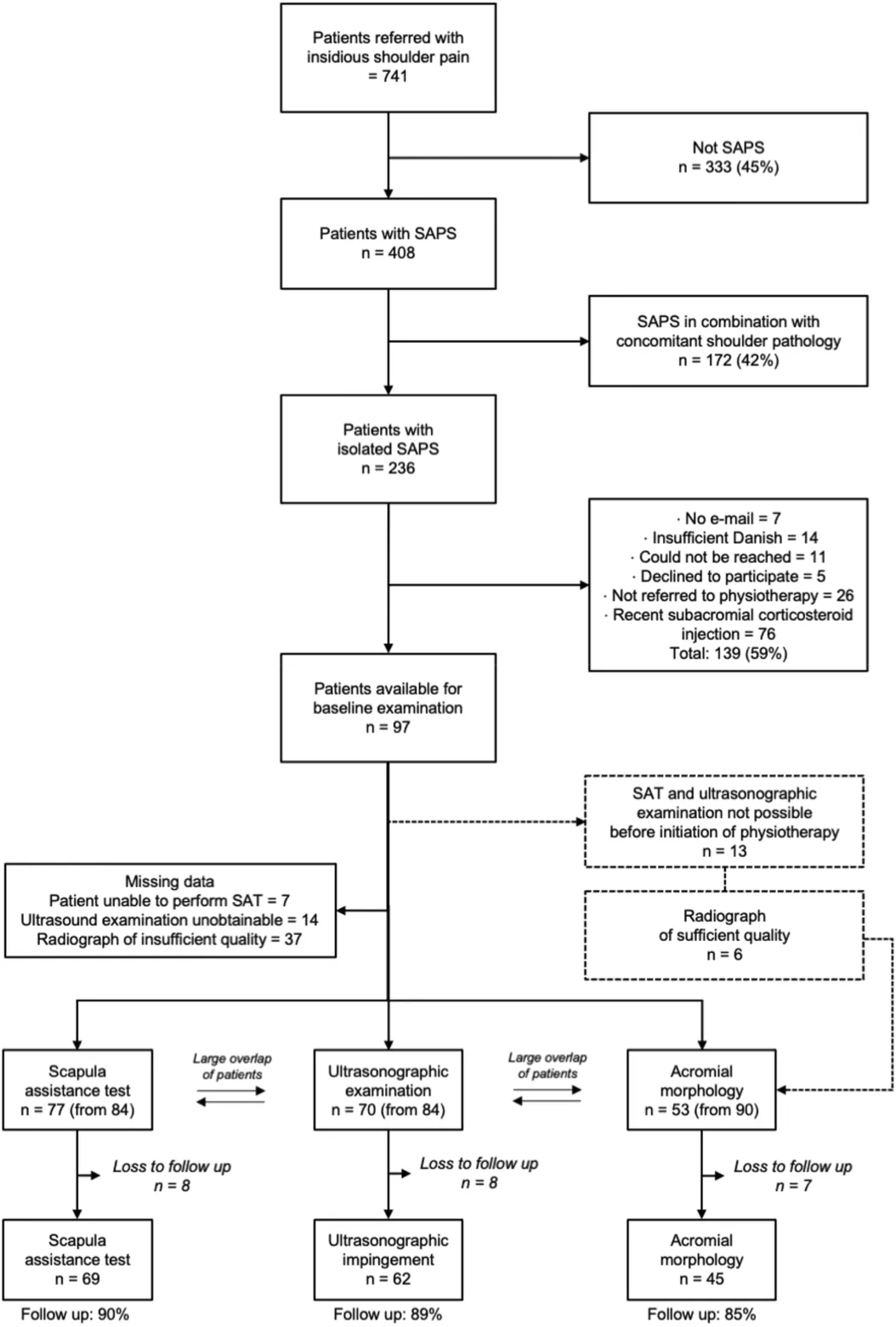
Patient flow. SAPS, subacromial pain syndrome; SAT, scapula assistance test.

From a total of 741 eligible patients referred with insidious onset of shoulder pain, 408 (55%) were diagnosed with SAPS. From these, 172 (42%) had concomitant diagnoses such as acromioclavicular osteoarthritis, full‐thickness rotator cuff tears or calcified tendinopathy. In total, 236 (32%) of the eligible patients were diagnosed with isolated SAPS and thus included in the study. From these, 142 (60%) patients were excluded: recent subacromial injection (76, 54%), not referred to physiotherapy (26, 18%), insufficient Danish (14, 10%), could not be reached (11, 8%), no e‐mail adress (7, 5%), declined to participate (5, 4%). Of the remaining 97 patients, 13 (13%) patients were unable to complete the baseline examination before their first physiotherapy session commenced. Six of these thirteen patients had an outlet view of sufficient quality and were included in the radiographic and demographic analyses. The remaining 84 patients underwent baseline examination with ultrasonography and evaluation of scapular control in addition to shoulder radiographs. Seven (8%) patients could not abduct their arm a minimum of 90° to participate in the SAT, and 14 patients (17%) could not participate in the ultrasound examination, as the required active movement during the examination provoked too much pain. Of the patients undergoing baseline examination, 37 (41%) did not have a radiograph of sufficient quality in accordance with the Copenhagen Outlet View Criteria [28]. Eight of the 90 patients were lost to follow‐up (9%). A total of 69 patients were included in the analysis for SAT, 62 patients for ultrasound, and 45 patients for the radiographic analysis (Figure 4). There was a large overlap of patients between these analytical groups. Accordingly, all the patients included in the ultrasound analysis and 89% of the patients included in the radiographic analysis were included in the SAT analysis. Demographics and descriptive statistics of pathophysiological factors are presented in Table 3.

**Table 3:** Demographics and pathophysiological factors.

Total number of patients	90
Age	55.5 (SD ± 12.2) (95% CI: 52.9–58.02)
Women	62%
BMI	26.9 (SD ± 5.2) (95% CI: 25.8–27.9)
Dominant shoulder affected	66%
Symptom duration	36 (SD ± 45) months; median: 18 months
SPADI, baseline	54 (SD ± 23) (95% CI: 49–59)
SPADI, 3‐month follow‐up	42 (SD ± 21) (95% CI: 38–46)
Acromial morphology *N* = 45	27.9° (SD ± 6) range: 18°–43°
Ultrasonographic impingement *N* = 62	Present: 50; Not Present: 12
Scapula assistance test *N* = 69	Positive: 43; negative: 26

*Note*: Values are presented as mean, unless stated otherwise.

Abbreviations: BMI, body mass index; CI, confidence interval; SD, standard deviation; SPADI, Shoulder Pain and Disability Index.

Acromial morphology was associated with poorer non‐surgical treatment outcome in both the adjusted and unadjusted analyses (Table 4). The adjusted analysis predicted a change in SPADI of 1.8 points per degree of increased acromial curvature (more hook‐shaped acromion). Neither scapular control nor ultrasonographic impingement was significantly associated with the change in SPADI There was no significant association between scapular control or ultrasonographic impingement to the change in SPADI after non‐surgical treatmentoutcome (Table 4). The mean time between completion of SPADI at baseline and follow‐up was 101 days.

**Table 4:** Association between pathophysiological factors and change in SPADI.

Pathophysiological factor	Change in SPADI	95% CI		*p*‐value	Effect size Cohen's *d*	*R* ^2^
		Lower limit	Upper limit			
Acromion morphology						
Unadjusted model	−1.4	−2.6	−0.3	0.015	≈0.72	0.12
Final model*	−1.8	−3.4	−0.25	0.026	≈0.70	0.11
*Interpretation: More‐hook shaped acromion leads to worse outcome (1.8 point change in SPADI per degree) after three months of non‐surgical treatment*						
Ultrasonographic impingement						
Difference between patients with and without ultrasonographic impingement	−3.5	−15.3	8.2	0.256	0.24	0.006
*Interpretation: Patients with ultrasonographic impingement have 3.5 points worse SPADI score after three months of non‐surgical treatment than patients without impingement (n.s.)*						
Scapular control						
Difference between *positive* and *negative* test	5.96	−3.9	15.6	0.137	0.29	0.02
*Interpretation: Patients with a positive SAT have 5.96 points worse SPADI score after three months of non‐surgical treatment than patients with negative SAT (n.s.)*						

Abbreviations: CI, confidence interval; SD, standard deviation; SPADI, Shoulder Pain and Disability Index.

* Final model adjusted for symptom duration, with longer duration associated to poorer outcome.

## DISCUSSION

In this exploratory study we found that an increased acromial curve (more hook‐shaped acromion) was associated with poorer outcome after 3 months of non‐surgical treatment. For each degree of increased acromial curvature, the adjusted model predicted a worse SPADI score of 1.8 points. As the MCID for SPADI has been reported to be 18 points [51], a 10° change in acromial curvature may approach clinical relevance regarding treatment outcome. Neither the presence of ultrasonographic impingement nor a positive SAT was associated with a significant change in SPADI after 3 months of non‐surgical treatment.

Acromial morphology varies considerably among patients, in a continuum ranging from a flat to a hook‐shaped undersurface [28, 34]. The latter has generally been theorised to be associated with SAPS. This theory was popularly introduced in 1972 by Charles S. Neer II in which he hypothesised that SAPS was caused by a curved/hook‐shaped acromion narrowing the subacromial space and leading to impingement of the subacromial structures [32, 33]. Yet, the association between acromial morphology and SAPS is poorly understood [15], and even less so the relationship between acromial morphology and non‐surgical treatment outcome, which has only been sparsely investigated [31, 45]. In a retrospective study, Morrison et al. reported a higher chance of satisfactory non‐surgical treatment outcome in patients with a type 1 acromion (a “flat” acromion according to the Bigliani classification [3]) compared to patients with type 2 (curved) or 3 (hooked), but no difference between patients with a type 2 and 3 acromion [31]. However, they did not adjust analyses for symptom duration. Wang et al. reported a higher risk of failed non‐surgical treatment in patients with a curved or hook‐shaped acromion compared to patients with a flat acromion, and found the highest risk of failure in patients with a hook‐shaped acromion [45]. The findings of the present study are consistent with those of Wang et al. and Morrison et al. However, the present study focused exclusively on patients with isolated SAPS, while Wang et al. and Morrison et al. included a broader patient population, not accounting for concomitant diagnoses such as acromioclavicular osteoarthritis or rotator cuff tears. Thus, a more detailed comparison between the present study and those of Wang et al. and Morrison et al. cannot be made. The findings of the present study add to the sparse literature, suggesting that a curved or hook‐shaped acromion may be negatively associated with non‐surgical treatment outcome. However, it must be emphasised that the present study is exploratory and that further studies are needed to confirm this association.

Acromial morphology is conventionally assessed on outlet view radiographs. However, even small deviations from an ideally projected outlet view have been shown to affect the perceived acromial morphology markedly [40]. In this study, only ideally projected outlet view radiographs, in accordance with the Copenhagen Outlet View Criteria [28], were included. 45 (50%) of the outlet views did not meet the predefined quality criteria for an ideally projected radiograph and were excluded from the analyses. Inclusion of outlet views with slight malrotation would have resulted in a higher sample size but could have compromised the validity of the findings. As all radiographs were obtained by radiographers blinded to the study and required no active shoulder movement from patients, the exclusion of suboptimal images is unlikely to have introduced sampling bias.

In the present study, the acromial curvature ranged from 18° to 43°, meaning that no patient had a “flat” acromion. This is an interesting finding that stands in contrast to previous studies reporting 8%–50% of patients with SAPS as having a “flat” (type 1) acromion [19, 35, 45]. However, it is important to note that these previous studies used the Bigliani classification which has been shown to be unreliable for evaluating acromial morphology [20, 28, 41]. The contrasting findings could also reflect differences in patient populations or the use of a continuous measurement scale in the present study, as a slight curvature may be classified as a “flat acromion” within the Bigliani classification.

Impingement is a dynamic phenomenon that can be observed with ultrasonography, primarily when the arm is elevated and internally rotated [6, 7]. The existing literature regarding impingement as an observable phenomenon is limited with a scarcity of documentation and a lack of consensus regarding its precise definition [6, 7, 15]. The difficulties associated with the lack of definition pose a challenge for investigations pertaining to its potential associations. In this study, a reliable and standardised method for evaluation of ultrasonographic impingement was used [24]. To our knowledge, no prior study has investigated ultrasonographic impingement in relation to non‐surgical treatment outcome in patients with SAPS. In this study, no significant association between ultrasonographic impingement and non‐surgical treatment outcome was found. Given the limited available evidence, more studies are required to fully understand whether, and through what mechanism, impingement influences treatment outcome.

Poor scapulothoracic control has been theorised to be an important pathophysiological factor for the development and maintenance of SAPS [23]. This theory is supported by the findings of a recent systematic review documenting the effect of scapular stabilisation exercises in reducing symptoms in patients with SAPS [52]. However, the direct influence of scapular control on treatment outcome is not understood. To our knowledge, this is the first study to investigate this. In this study, we found no significant association between SAT and non‐surgical treatment outcome. Interestingly, most patients had a positive SAT (62%). While scapular dysfunction is evidently common in SAPS, its role in predicting non‐surgical treatment outcome does not seem straightforward. The findings of this study do not rule out scapular control as a potentially important factor in the non‐surgical management of SAPS, as the interplay between scapular movement and shoulder pain may be too complex to assess with a single clinical test. A more comprehensive evaluation may be necessary to fully understand its contribution

It should be noted that a modified SAT was used in the present study, incorporating an additional range‐of‐motion criterion, which limits comparability with previous studies. The modification was implemented to address the expectation that some patients would be unable to fully abduct their arm due to severe shoulder pain, while recognising that a substantial increase in abduction during the SAT (e.g., from 90° to 180°) at the same pain level would still represent a clinically meaningful improvement. Such ROM improvements would not have been captured using the original SAT.

### Strength and weaknesses

The inclusion of a homogenous population with isolated SAPS identified through a systematic and standardised setup is a key strength of the study that offers favourable conditions for investigating fundamental pathophysiological factors. The standardised and protocolized physical examination tests and systematic screening for concomitant shoulder diagnoses increase the reproducibility of the findings.

The study utilised a pragmatic approach adhering to the Danish national guideline for treatment of SAPS. This pragmatic approach reflects the current clinical practice but also introduces risk of bias. A considerable portion of the eligible patients were excluded, most of them from not being referred to physiotherapy or or having received a recent subacromial corticosteroid injection. Possible reasons for patients not being referred to physiotherapy could be that the patients had undergone a recent physiotherapy programme before their referral or that physiotherapy was deemed unnecessary, perhaps due to mild symptoms. This restricts generalisability to those where physiotherapy is deemed necessary. A sampling bias could also have occurred from excluding patients who have had a recent subacromial corticosteroid injection as these patients might have had more severe symptoms. Additionally, some of the included patients had symptoms too severe to participate in the ultrasonographic examination (*n* = 14 (7%)) and the scapular evaluation (*n* = 7 (8%)), which may similarly represent a similar sampling bias from excluding patients with more severe symptoms.

It should be noted that the physioherapy protocol was not standardised. This was not deemed feasible due to a considerable variation in symptom severity among patients. While some patients were able to perform weighted exercises, others struggled to lift their arm and typically began with unweighted or passive exercises. Patients experiencing prolonged post‐exercise pain were most often advised to skip subsequent sessions. While this approach reflects real‐world conditions, it also resulted in substantial heterogeneity in the physiotherapy patients underwent. Compliance was not included in the analyses as this could have led to skewed results.

### Implications for further research

In this study, hypothesised pathophysiological factors were explored for possible associations to treatment outcome. Following the PROGnosis RESearch Strategy (PROGRESS) framework [21], this represents the second step (out of four): the identification of factors that are associated with prognosis (prognostic factor research). Of the explored pathophysiological factors, acromial morphology was identified as a potential prognostic factor. Given the limited evidence on acromial morphology as a prognostic factor, and in accordance with the PROGRESS framework, future research should initially seek to replicate and confirm the findings of the present study [37]. Future studies should consider using the CAC classification over the traditionally more used Bigliani classification, given its higher reliability. Furthermore, the CAC may be more useful for establishing clinically relevant cut‐off points as its continuous scale facilitates the use of more advanced statistical analyses, in contrast to the ordinal‐scale Bigliani classification. Future multicenter prospective studies should confirm acromial morphology as a prognostic factor and investigate whether morphology‐based stratification has the potential to improve outcomes.

## CONCLUSION

In patients with isolated SAPS, increasing acromial curvature (more hook‐shaped acromion) was associated with poorer non‐surgical treatment outcome, while ultrasonographic impingement and scapular control were not. This indicates that acromial morphology may be a prognostic factor for non‐surgical treatment outcome, but future research is needed to confirm the findings.

## AUTHOR CONTRIBUTIONS

Adam Witten, Per Hölmich and Kristoffer Weisskirchner Barfod conceived the study and designed the methodology. Adam Witten collected and curated the data. Adam Witten performed the statistical analysis and interpretation of results. Kristoffer Weisskirchner Barfod assisted in the interpretation of results. Adam Witten drafted the initial manuscript. Adam Witten, Kristian Thorborg, Mikkel Bek Clausen, Per Hölmich and Kristoffer Weisskirchner Barfod critically revised the manuscript for important intellectual content. All authors read and approved the final version of the manuscript.

## CONFLICT OF INTEREST STATEMENT

The authors declare no conflicts of interest.

## ETHICS STATEMENT

The study was registered and approved by the Regional Scientific Ethical Committee, Copenhagen Region (Reference no.:H‐19025712). The protocol was uploaded to ClinicalTrials.gov (NCT05549674).

## Supporting information


Supporting File

## Data Availability

The data that support the findings of this study are available on request from the corresponding author. The data are not publicly available due to privacy or ethical restrictions.
