# Supplementary material for: Association between acromial morphology, scapular control, ultrasonographic impingement and non‐surgical treatment outcome in patients with isolated subacromial pain syndrome
Source: Knee Surg Sports Traumatol Arthrosc. 2026 May 20;34(9):3429–40. doi: 10.1002/ksa.70424 (PMC13502451; doi:10.1002/ksa.70424)
Supplement: Supplementary file 1 [file KSA-34-3429-s001.docx]

**APPENDIX

Detailed description of physical examination tests**

Hawkins’ test
The examiner places the patient’s shoulder in 90 degrees abduction with the elbow into 90 degrees flexion, in the scapular plane. The examiner internally rotates the patient’s shoulder. The test is positive if recognizable shoulder pain is reproduced.

Neer’s test
The examiner places one hand on the back and top of the patient’s shoulder (to avoid scapular protraction and external rotation). With the other hand, the examiner then flexes the patient’s shoulder from 0 to 180 degrees. The test is positive if recognizable shoulder pain is reproduced.

Jobe’s test
The patient is instructed to put both shoulders in 90 degrees flexion in the scapular plane and in internal rotation (thumbs pointing down). The patient is instructed to hold the position while the examiner applies downward force on the patient’s forearms. The test is positive if recognizable shoulder pain is reproduced.

Painful Arc
The patient is instructed to abduct the shoulder in the coronal plane. The test is positive if recognizable shoulder pain is reproduced with maximum pain experienced between 60 to 120 degrees abduction.

External rotation resistance test (resisted isometric external shoulder rotation)
The patient is instructed to flex both elbows to 90 degrees. The patient is thee instructed to hold the position while the examiner applies (internal rotational) force on the patient’s distal forearms. The test is positive if recognizable shoulder pain is reproduced.

Apprehension test
The patient is lying supine. The shoulder is abducted to 90 degrees with the elbow in 90 degrees flexion. Using the forearm as a lever, the examiner externally rotates the patient’s shoulder forcing the humeral head anteriorly. The test is positive when the patient demonstrates an apprehensive feeling. The test is negative if the patient only experiences pain.

Relocation test
The patient is lying supine. The shoulder is abducted to 90 degrees with the elbow in 90 degrees flexion. Using the forearm as a lever, the examiner externally rotates the patient’s shoulder forcing the humeral head anteriorly. The examiner then applies a posterior-directed force on the humeral head. If this relieves an apprehensive feeling, the test is positive.

Surprise test
From the end position of the relocation test, the posterior-directed force on the humeral head is removed quickly without preparing the patient (examiners hand is removed from the patient’s shoulder). The test is positive if the patient demonstrates an apprehensive feeling.

Castagna’s test
Same position as for Jobe’s apprehension test, but with the arm abducted to 45 degrees instead of 90. The test is considered positive if the patient reports shoulder pain.

Jerk test
The patient is sitting comfortably on a chair. The examiner stabilizes the patient’s scapula and positions the patient’s arm in 90 degrees abduction and internal rotation. From this position the examiner applies longitudinal pressure on the humerus (pressure on patient’s elbow) while moving the arm horizontally across the patient’s body (horizontal adduction). The test is considered positive if the humeral head slides up on the glenoid rim (sudden clunk) or if pain is felt.

O’Brien’s test
The patient is instructed to abduct the shoulder 90 degrees and adduct the shoulder 10 degrees. With the patient’s shoulder in slight internal rotation (thumbs up), the patient is instructed to keep the position, while examiner applies downward pressure on the patient’s forearm. The patient then brings the shoulder into further internal rotation (thumbs down). The patient is instructed to keep the position, while the examiner applies downward pressure on the patient’s forearm. The test is considered positive if the patient reports pain in the second position (thumbs down).

Speed’s test
With the shoulder resting in external rotation, the patient is instructed to elevate the arm (shoulder flexion), while the examiner applies pressure on the patient’s forearm throughout the movement. The test is considered positive if the patient reports shoulder pain in the bicipital groove.

Long head biceps tendon palpation pain

The patient stands in a relaxed position with palms facing forward. The examiner palpates the long head biceps tendon in the sulcus. The test is considered positive if the patient reports pain at the site of palpation.

Cross-over test
The examiner brings the patients shoulder into 90 degrees of flexion and then maximum adduction. The test is considered positive if the patient reports pain at the acromioclavicular joint.

Acromioclavicular joint palpation pain

The examiner palpates the superior aspect of acromioclavicular joint. The test is considered positive

if the patient reports pain at the site of palpation.

**Detailed description of exclusion criteria**

Frozen shoulder
Frozen shoulder was defined as a progressive equal loss of active and passive shoulder ROM greater than 25 degrees in at least two planes, and a loss of external rotation greater than 50% when compared to the contralateral (unaffected) shoulder.

Glenohumeral osteoarthritis
Osteoarthritis of the glenohumeral joint was defined as radiological evidence of glenohumeral osteoarthritis (Kellgren and Lawrence score of 3 or 4).

Previous surgery
Includes all surgical procedures in the shoulder girdle.

Fractures
Includes fracture of the proximal humerus, clavicle and scapula (including osseous Bankart lesions). Hill Sachs lesion was not an exclusion criterion.

Radiotherapy
Includes previous radiotherapy in the shoulder girdle, neck or upper thoracic region.

Cervical pathology
A positive Spurling test (foramen compression test).

**Inter-rater reliability of the Scapula Assistance test**
Consecutive patients with subacromial pain from an orthopedic outpatient clinic were eligible for inclusion if they had at least three out of five positive tests from the following: Hawkin’s, Neer’s, Jobe’s, Painful Arc and External Rotation Resistance Test. A medical student and an orthopedic resident (first author) performed the Scapula Assistance test (rated positive or negative). The two raters were blinded to each other’s results. 33 patients (mean age: 52 years, SD: 19) were included during a three-month period. The overall agreement for SAT was 82%, and Kappa was 0.61.
